# Studies on the Transcorrelated Method

**DOI:** 10.1021/acs.jctc.3c00046

**Published:** 2023-08-28

**Authors:** Nicholas Lee, Alex J. W. Thom

**Affiliations:** † Department of Chemistry, Physical and Theoretical Chemistry Laboratory, South Parks Road, Oxford OX1 3QZ, U.K.; ‡ Yusuf Hamied Department of Chemistry, Lensfield Road, Cambridge CB2 1EW, U.K.

## Abstract

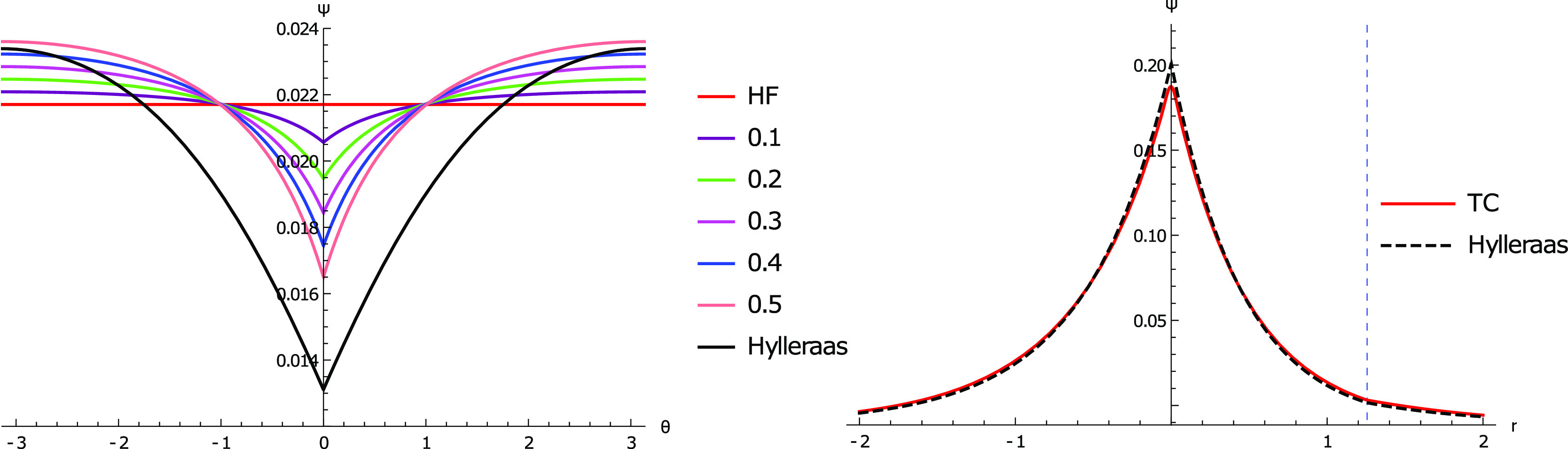

We investigate the possibility of using a transcorrelated (TC) Hamiltonian to describe electron correlation. A method to obtain TC wavefunctions was developed based on the mathematical framework of the bi-variational principle. This involves the construction of an effective TC Hamiltonian matrix, which can be solved in a self-consistent manner. This was optimized using a method we call second-order-moment minimization and demonstrate that it is possible to obtain highly accurate energies for some closed-shell atoms and helium-like ions. The effects of certain correlator terms on the description of electron–electron and electron–nuclear cusps were also examined graphically, and some TC wavefunctions were compared against near-exact Hylleraas wavefunctions.

## Introduction

Capturing the effects of electron correlation is a central problem in electronic structure theory. A possible approach to tackle the problem involves the use of a similarity transformed Hamiltonian , where the τ is a polynomial dependent on electronic positions and incorporates explicitly the correlation between various electron pairs. The use of such a Hamiltonian is known as the transcorrelated (TC) method. The inclusion of *r*_12_ terms to describe electronic correlation can be dated back to Hylleraas^1^ and later popularized by Kutzelnigg,^2^ forming the basis of R12/F12 methodology^3,4^ today. Boys and Handy employed the TC formalism to introduce correlation terms to get near-exact energies for various atoms and molecules.^5−11^ This was done using a custom basis set and an optimized Jastrow factor. Hirschfelder,^12^ Bartlett and Nooijen,^13^ Klopper and co-workers,^14,15^ and Ten-no^16−21^ have also considered the use of such similarity-transformed Hamiltonians to eliminate the singularities associated with the  term in the many-electron Hamiltonian.

The TC method joins in the family of explicitly correlated methods^22−24^ and is closely related to canonical TC theory^25,26^ and F12 methods. In canonical TC theory, the similarity transformation of the molecular Hamiltonian is not unitary. The formal difference leads to different approximations being made. F12 methods have now found widespread use in providing highly accurate electronic structure calculations. F12 methods employ geminal functions formed from the application of a rational generator (to impose cusp conditions) and a strong orthogonality projector on a two-electron determinant. The TC method attempts to recover dynamic correlation similarly by introducing cusp conditions and *r*_12_ terms through its Jastrow factor. This represents a straightforward approach to considering the problem of explicitly correlated electrons.

There are two principal difficulties working with the TC Hamiltonian. First, the TC Hamiltonian will involve three-electron operators, which can be expensive computationally. Second, the TC Hamiltonian is non-Hermitian. Unlike with Hermitian operators, the variational principle does not hold for non-Hermitian operators; hence, unphysical energies below that found from full configuration interaction (FCI) may be obtained. Furthermore, non-Hermitian matrices are difficult to work with due to the possibility for numerical instability in matrix computations.

However, with the introduction of variational Monte Carlo (VMC), the calculation became more computationally feasible and promising results were shown for a variety of atoms, molecules,^15,27−29^ and periodic systems.^30,31^ To tackle the issue of non-Hermiticity, Luo proposed to replace the non-Hermitian ansatz with a Hermitian approximation so that a variational approach becomes viable.^32,33^

The TC Hamiltonian has also more recently been used with a variety of quantum chemistry methods with promising results. Alavi and co-workers have applied FCI quantum Monte Carlo (FCIQMC) to the TC ansatz for a variety of systems successfully.^34−37^ Their numerical results show that the unboundedness of the non-Hermitian operator did not pose serious difficulties and that highly accurate results, even up to spectroscopic accuracies,^38^ could be realized. More recently, they have used the TC Hamiltonian with coupled cluster,^39^ and the energies found demonstrated better basis set convergence. On the other hand, Reiher and co-workers have developed a TC analogue of Density Matrix Renormalization Group and applied it to the Fermi-Hubbard model and some homo-nuclear diatomics.^40,41^ They have similarly found that the use of transcorrelation accelerates the convergence to the complete basis set limit.

Building on the recent successes of the TC method, this work shall attempt a deterministic approach and examine its viability as a computational tool to capture the effects of electron correlation. While Boys and Handy’s formulation was deterministic, the computational resources of their time would have restricted the scope of their work. We therefore think that it would be useful to explore the possibilities of a deterministic approach with the computational resources today. Unlike Boys and Handy, however, we will solve the non-Hermitian Hamiltonian matrix self-consistently. Instead of parametrizing the correlator using the contraction equations, we propose to find it via what we shall call second-order-moment (SOM) minimization, an analogue of variance minimization that we have adapted for this work.

## Theoretical Background

### TC Hamiltonian

Given a Slater determinant Φ, Boys and Handy suggested^5^ that the eigenfunction of the many-electron Hamiltonian can be approximately written as Φ_TC_ = e^τ^Φ. τ is a function which accounts for electron correlation and is known as the correlator, while e^τ^ is commonly referred to as the Jastrow factor. From the Schrödinger equation
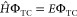
1
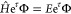
2
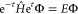
3
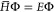
4In the final step, we make the identification . This is the TC Hamiltonian. The TC Hamiltonian can be expanded using the Baker–Campbell–Hausdorff expansion
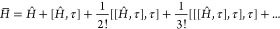
5By using a correlator of the form τ = ∑_*i<j*_*u*(***r***_*i*_, ***r***_*j*_), the third- and higher-order commutator terms vanish. The commutators can be further expanded to give
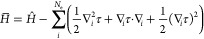
6where *N*_e_ is the total number of electrons in the system studied.

Substituting τ = ∑_*i<j*_*u*(***r***_*i*_, ***r***_*j*_), the TC Hamiltonian takes the form
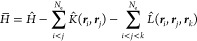
7where
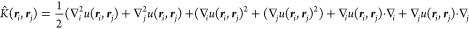
8
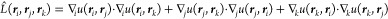
9The presence of the terms **∇**_*i*_*u*(***r***_*i*_, ***r***_*j*_)·**∇**_*i*_ + **∇**_*j*_*u*(***r***_*i*_, ***r***_*j*_)·***∇***_*j*_ in  makes the TC Hamiltonian non-self-adjoint. This has been derived previously in several papers^6,14,15,35^ but is recapitulated here for completeness.

### One-Electron Effective Hamiltonian

The TC Hamiltonian is non-self-adjoint and will therefore have left- and right-eigenvectors. The left- and right- eigenvectors  and  are Slater determinants formed from molecular orbitals {ψ_1_ψ_2_...ψ_*n*_} and {ϕ_1_ϕ_2_...ϕ_*n*_}, respectively. The Slater determinants satisfy the following equations
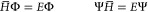
10with *E* denoting the energy associated with the eigenvectors. A bi-orthonormal set of molecular orbitals, that is, ⟨ψ_*i*_|ϕ_*j*_⟩ = δ_*ij*_ can always be found via Löwdin pairing^42^ and hence we assume bi-orthonormality throughout this paper.

The TC energy can be identified as
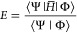
11
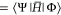
12

The denominator is unity due to the bi-orthonormality condition. The effective TC Hamiltonian can be found by taking the functional variation of the TC energy. The functional variation can be found by using the method of Lagrange multipliers. Forming the Lagrangian  under the constraint of bi-orthonormal orbitals
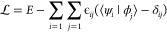
13

We seek the solution to  to find a stationary point of the energy with respect to the constraint. We prove in the Appendix that using the condition , we get the equation
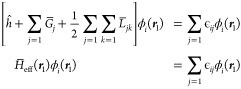
14such that
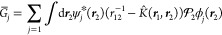
15
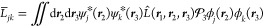
16

We also introduce a notation . *S*_N_ is the symmetric group of degree *N*. For example
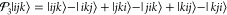
17 therefore gives all the possible permutations (with the correct parity) of the three-particle ket |*ijk*⟩.  is the effective TC Hamiltonian. It is a functional of the bi-orthogonal set of molecular orbitals {ψ_*i*_} and {ϕ_*i*_} and can thus be solved for iteratively through a self-consistent approach.

### Jastrow Factor

The following form of the correlator was first introduced by Boys and Handy^8^
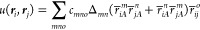
18where
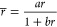
19and
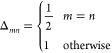
20

Scaling of the inter-particle distances as *r̅* is known as the Padé form.^43^ Scaled distances are commonly used for Jastrow factors such that at large inter-particle distances, the terms in the Jastrow factors will approach a constant. There have been a number of scaling functions employed in literature.^44^ Following the work of Schmidt and Moskowitz,^45,46^ we will use the Padé form with *a* = *b* = 1 due to the simplicity of implementation.

### Optimizing Correlator Parameters

The correlators are a function of the set of parameters {*c*_*mno*_}. However, determination of these parameters is a non-trivial task. While the parameter *c*_001_ in eq 18 has been determined previously to be 1/2 to satisfy the cusp condition, the other parameters *c*_*mno*_ have yet to be determined. The unbounded nature of the non-self-adjoint TC Hamiltonian operator prevents the use of energy minimization for this. However, minimization of the local energy variance can be performed to find these parameters. Schmidt and Moskowitz applied VMC to calculate and minimize the variance. They performed this with correlators consisting of 7, 9, and 17 terms and found that with a 17 term correlator, 68–100% of the correlation energies for atoms helium through neon could be recovered using their variance-minimized parameters.

Handy also independently developed a variance minimization procedure to optimize the TC parameters.^47^ He introduced the TC variance
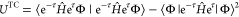
21

The minimization of *U*^TC^ was performed through the Davidson method and near-exact energy for the helium atom was calculated through this method, albeit with a slight modification of the Jastrow factor. However, helium is a two-electron system; for any systems with more than two electrons, the three-electron operator in the TC Hamiltonian will in general give a non-zero term. As such, the calculation of the TC variance in eq 21 will require the evaluation of six-electron operators. The high computational cost and poor scaling has deterred research efforts along this line of inquiry.

### SOM Minimization

While variance is well-defined for a self-adjoint operator, there is little literature for its non-self-adjoint counterpart. It is well known from linear algebra that a non-Hermitian matrix has left- and right-eigenvectors, which are not necessarily identical. The TC Hamiltonian is a non-self-adjoint operator and would similarly have left- and right-eigenfunctions Ψ and Φ, respectively. We assume the use of a bi-orthogonal basis such that ⟨Ψ|Φ⟩ = 1. Instead of variance minimization, we propose the minimization of the SOM, a bi-orthogonal analogue of the variance for a non-self-adjoint Hamiltonian
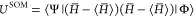
22where . This is a bi-orthogonal extension to the usual definition of the variance (or second central moment in some papers^48,49^). To the best of the authors’ knowledge, the minimization of *U*^SOM^ has not previously been performed. We shall first analyze some limiting cases to gain a better understanding of the quantity *U*^SOM^.

In the limit of  (self-adjointness), the left- and right-eigenfunctions become identical, Ψ = Φ. *U*^SOM^ therefore reduces to the standard definition of the variance
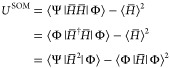
23In the limit that Φ is an exact eigenfunction, that is, *H̅*Φ = λΦ where 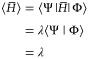
24where we have made use of the bi-orthogonality of the left- and right-eigenfunctions. Then
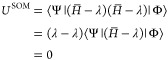
25

A similar proof holds for the limit that Ψ is an exact eigenfunction. Since the exact eigenfunction has to satisfy the condition that *U*^SOM^ = 0, the parameters should be varied such that the quantity *U*^SOM^ becomes as close to zero as possible. The evaluation of *U*^SOM^ would similarly require the evaluation of six-electron terms (three from each *H̅*) and it is therefore as computationally challenging as Handy’s TC variance. To side-step this difficulty, the resolution of identity is employed. In the bi-orthogonal basis, the identity is given by
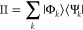
26where *k* runs through all of the possible Slater determinants for the given basis.
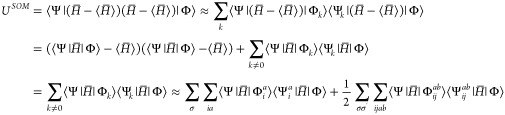
27where the factor of a half was added to take into account double counting of *ij* and *ab*. The σ terms denote the various spins of electrons. The penultimate step is an approximation as we ignore the triple excitation terms when they are much smaller than the double excitation terms. In addition, the single excitation term has terms with: . By analogy to Brillouin’s theorem for the Hartree–Fock method, single excitation determinants will not interact directly with the ground-state determinant, that is, . We can therefore ignore the single excitation terms and deduce that
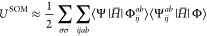
28

The use of RI approximation in the context of transcorrelation is not new. The RI approximation had been used to evaluate the three-electron terms efficiently.^18^ In this work, the approximation was used to evaluate the expectation value of the square of the TC Hamiltonian. Without any approximations, the expectation value of a six-electron operator would have to be evaluated, which would have been computationally impractical.

### Bi-Variational Principle

Having found the appropriate correlator parameters, we can construct the TC Hamiltonian and solve for its eigenfunctions. However, when the Hamiltonian is non-self-adjoint (as in the case for the TC Hamiltonian), the variational principle does not hold and the expectation value of the Hamiltonian is not bound from below. A naive minimization of the Hamiltonian’s expectation value can therefore lead to values below the exact ground state energy, which are unphysical. However, one can formulate a different variational principle for a generic operator, which is not necessarily self-adjoint. While the mathematical exposition on the bi-variational principle has been previously undertaken by Löwdin,^50−52^ the essential parts of the proofs are reviewed here as it is crucial to the development of the TC method.

For a non-self-adjoint operator *H̅*, we can define left and right eigenfunctions Ψ and Φ, respectively, such that
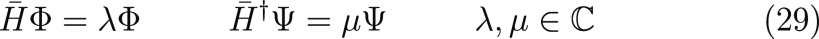
We note that λ and μ are related by complex conjugation, that is, λ = μ* For a given pair of trial functions Ψ_*i*_ and Φ_*i*_ such that
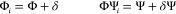
30the expectation values λ_*i*_ and μ_*i*_ are given by
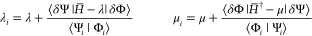
31

The expectation values λ_*i*_ and μ_*i*_ have vanishing first-order variations (δλ_*i*_ = 0), that is, they correspond to stationary points about the exact eigenvalues λ and μ, respectively. This is known as the *bi-variational principle for a pair of adjoint operators*.^50^

Conversely, we can show that if δλ_*i*_ = 0 for all δΦ and δΨ
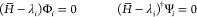
32

This implies that the trial function Φ_*i*_ is an eigenfunction of *H̅* with eigenvalue λ_*i*_ and Ψ_*i*_ is an eigenfunction of  with eigenvalue λ_i_^*^ = *μ*_*i*_. Equation 31 implies that if the trial functions Φ_*i*_ and Ψ_*i*_ are correct to the first order, the approximation of the eigenvalue λ_*i*_ to the exact eigenvalue λ is correct to the second order.

### Matrix Representation

The bi-variational equations can be recast in matrix form. In the following, the tensor notation of Head-Gordon et al.^53^ shall be used. Given an atomic-orbital basis {χ_1_...χ_*n*_}, we can expand any pair of trial functions Φ_*i*_ and Ψ_*i*_ as
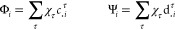
33From eq 32, the bi-variation equations can then be expressed as
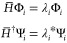
34
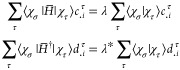
35
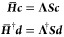
36

The expressions in eq 35 were obtained through left-multiplying by χ_σ_ and integrating over all space. In the last step, we make the identification that  and ***S***_στ_ = ⟨χ_σ_|χ_τ_⟩.

### Solving the TC Equation

We are now in a position to apply the bi-variational approach on the TC Hamiltonian. The effective TC Hamiltonian matrix has to be solved iteratively as the two- and three-electron terms are dependent on the trial functions Φ_*i*_ and Ψ_*i*_. The following workflow was utilized:

1. Perform Hartree–Fock calculation and use the Hartree–Fock coefficients as a starting guess.
2. Build the effective TC Hamiltonian matrix.
3. Diagonalize the matrix to get new coefficients for the left- and right-eigenvectors.
4. Repeat until convergence.

In doing so, we are simultaneously optimizing both the left- and right-eigenvectors. This is a different approach to that of Dobrautz, Luo, and Alavi^36^ where only the right-eigenvector is optimized. While our approach requires the optimization of both left- and right-eigenvectors, which translates to a more expensive calculation, we gain the benefit of bounding the error of the calculation by the bi-variational principle. The bi-variational approach proposed here shares similarity with that introduced by Ten-no.^18^ However, we treat the TC Hamiltonian in full, without approximations to the three-electron term. The inclusion of three-electron contributions to the Fock matrix is also in contrast to Ten-no’s approach^21^ where the three-electron term is omitted. The error from our calculations comes from the error in using numerical grids and not from an approximation in our working equations.

### Maximum Overlap Method

Convergence of the bi-variational approach can be difficult in some cases. Taking inspiration from the work of Gilbert and co-workers,^54^ we first assume that the Hartree–Fock coefficients are a good guess at our final coefficients. Therefore, at each iteration, the set of orbitals with the largest overlap to the occupied orbitals in the previous iterations will be picked. This process proceeds until convergence is reached. This is known as the maximum overlap method (MOM). Given the right coefficient matrix from the previous iteration ***C***_old_, the left coefficient matrix from the current iteration ***D***_new_, and the atomic orbital overlap matrix ***S***, the maximum overlap matrix ***O***_MOM_ is given by
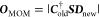
37

The bi-orthogonal solutions from each iteration are determined only up to a phase factor, and hence the modulus is taken to ensure that the overlap remains positive.

Even with traditional implementations of MOM, it is found that it is possible for self-consistent field (SCF) iterations to converge onto unwanted solutions. This has led to the introduction of the initial MOM (IMOM),^55^ where new orbitals in each iteration are picked based on their overlaps with the initial guess orbitals. This prevents the solutions from drifting away from the initial guess and has been shown to give better convergence to desired solutions. In this work, we adapt it for bi-orthogonal orbitals, such that the maximum overlap matrix ***O***_IMOM_ is given by
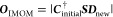
38where ***C***_initial_ is the initial left coefficient matrix.

Both forms of the MOM were implemented for improved convergence of the iterative procedure.

## Computational Details

The TC method is implemented in Python. The matrix elements relating to the correlator were found via numerical integration. These integrations were performed with grids found in the PySCF package. Throughout this work, we used Treutler–Ahlrichs grids with Becke partitioning. Q-Chem 5.3 was used for conventional non-orthogonal configurational interaction (NOCI) calculations and for finding Hartree–Fock solutions. Mathematica was used to plot Figures 2–8.

**Figure 1:**
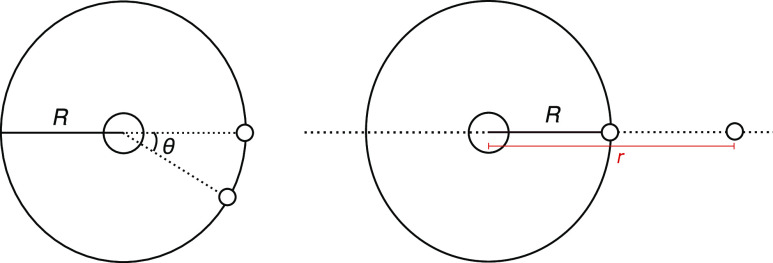
(Left) two electrons constrained at a fixed electron–nuclear distance of *R*. The electron–electron distance is modulated by their angle of separation, θ. θ is measured in radians. (Right) one electron is fixed at distance *R* and the other is constrained to the (dotted) line defined by the nucleus and the fixed electron. *R* is found by the expectation value of the electron–nuclear separation in near-exact wavefunction given by Nakashima and Nakatsuji and co-workers.^61^

**Figure 2:**
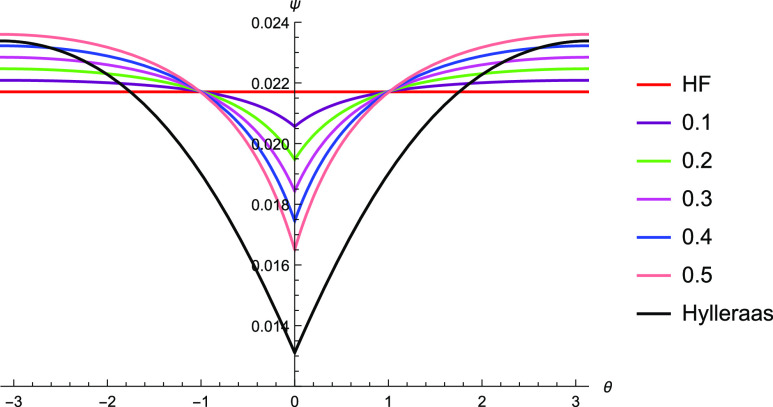
Graphical description of the electron–electron cusp of the TC wavefunction with *c* = 0.1 through *c* = 0.5 against Hartree–Fock and Hylleraas wavefunctions. The function with *c* = 0.5 most closely matches that of the Hylleraas wavefunction, but the cusp is shallow as compared to the Hylleraas wavefunction.

**Figure 3:**
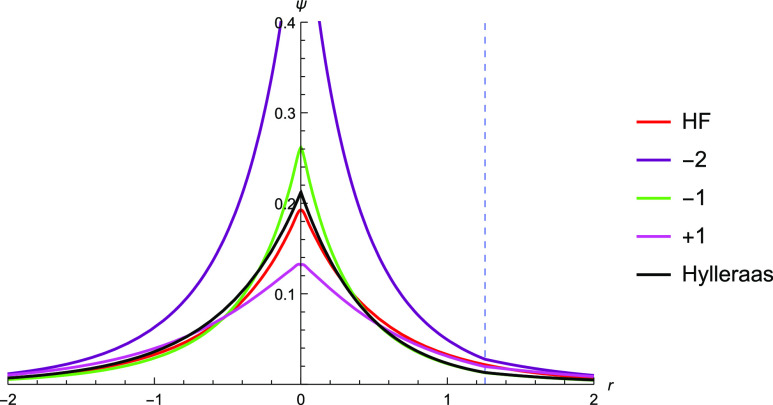
Graphical description of the electron–nuclear cusp of the TC wavefunction of He with *c* = −2, −1, and 1 against Hartree–Fock and Hylleraas wavefunctions. The position at which the other electron is fixed is shown by the dashed blue line.

**Figure 4:**
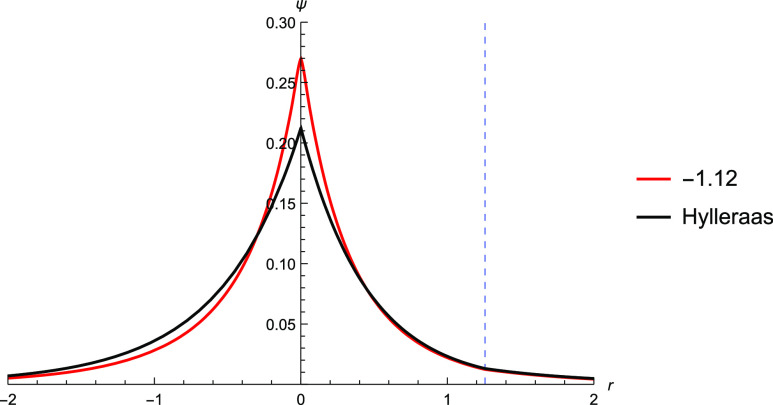
Plotting the Hylleraas wavefunction against the TC wavefunction with *c* = −1.12. *c* was found by SOM minimization and was performed with a cc-pV5Z basis set. The Slater determinant used is found via Hartree–Fock calculation using a cc-pVQZ basis. While there is some discrepancy between the TC wavefunction and the Hylleraas wavefunction near the nucleus, the two wavefunctions are very similar further away from the nucleus.

**Figure 5:**
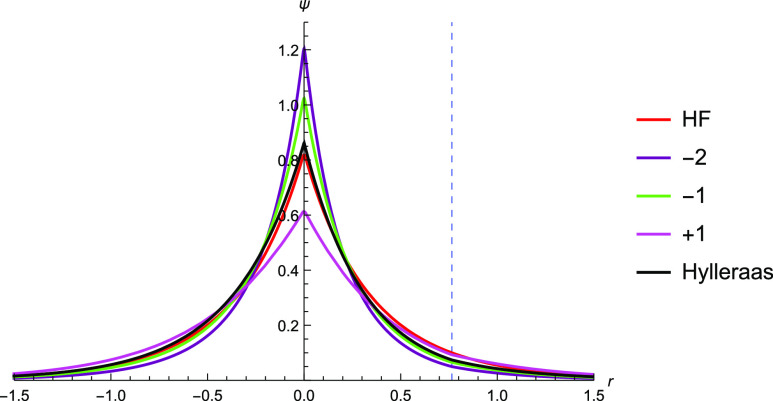
Graphical description of the electron–nuclear cusp of the TC wavefunction of Li^+^ with varying values of *c* against Hartree–Fock and Hylleraas wavefunctions. The Hartree–Fock wavefunction resembles the Hylleraas wavefunction near the nucleus. The TC wavefunction with *c* = −1 most closely matches that of the Hylleraas wavefunction further away from the nucleus.

**Figure 6:**
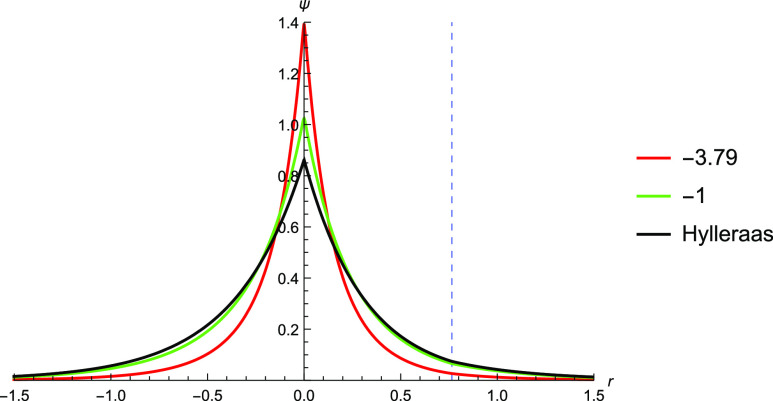
Plotting the Hylleraas wavefunction against the TC wavefunction with *c* = −3.79 and *c* = −1. *c* was found by SOM minimization with a cc-pCVQZ basis set. The Slater determinant used is found via Hartree–Fock calculation using a cc-pVQZ basis.

**Figure 7:**
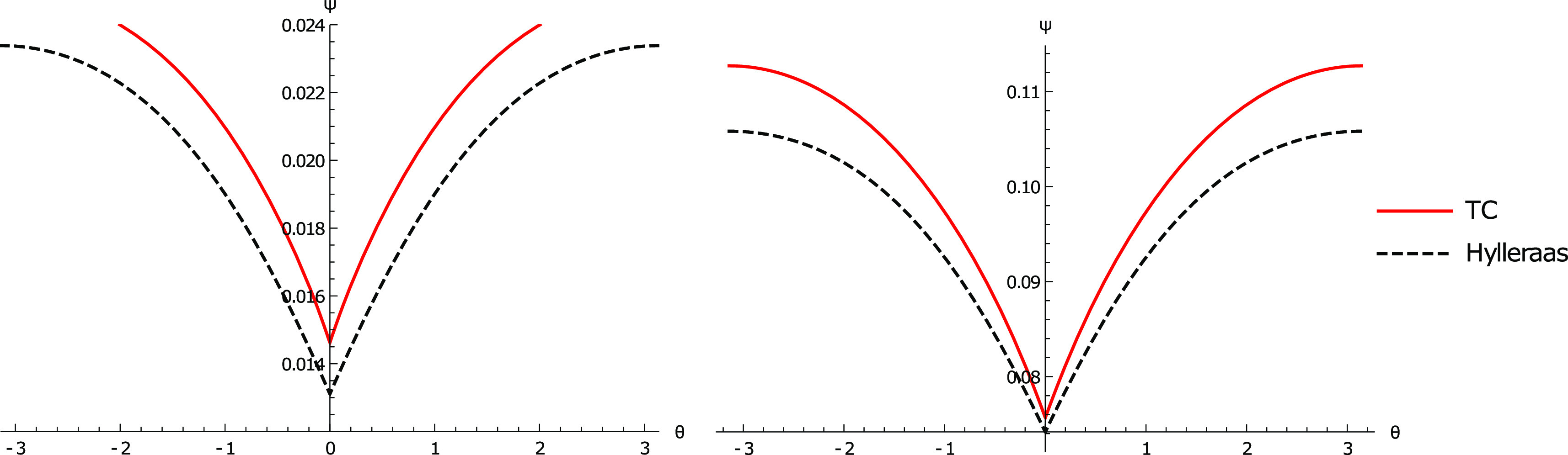
Two plots describing the electron–electron cusp corresponding to Figure 1 (Left). (Left) plot of the TC wavefunction for He against the corresponding Hylleraas wavefunction. (Right) plot of the TC wavefunction for Li^+^ against the corresponding Hylleraas wavefunction. Both plots show that the 18 parameter TC wavefunction reproduces the shape of the electron–electron cusp, but the cusp is shallower than the Hylleraas wavefunction.

**Figure 8:**
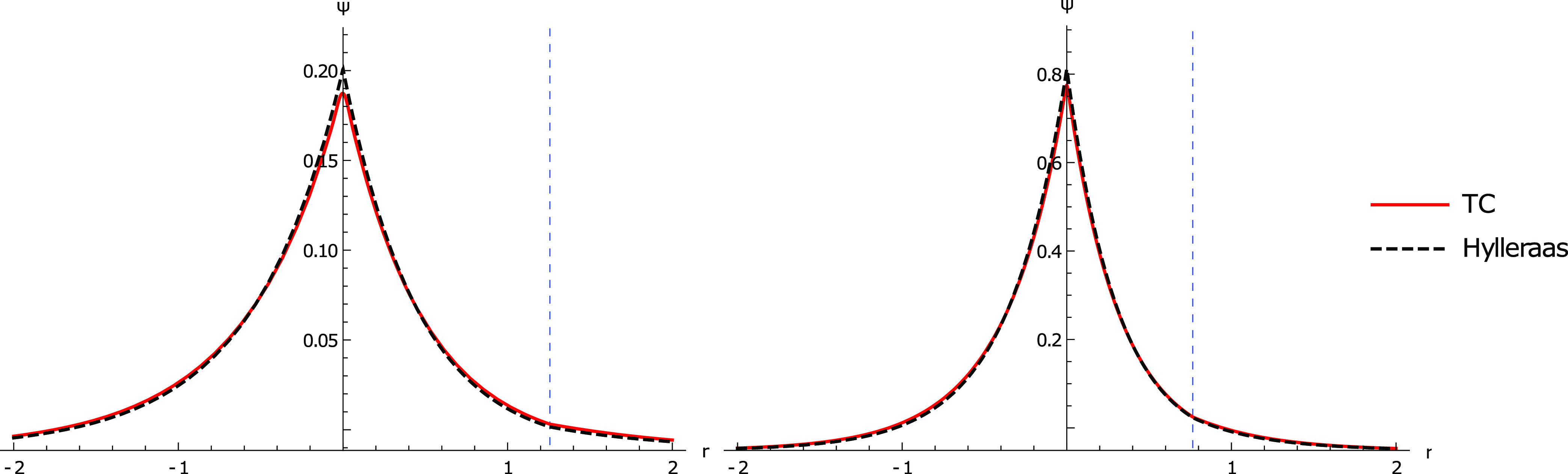
Two plots describing the electron–nuclear cusp corresponding to Figure 1 (Right). (Left) plot of the TC wavefunction for He against the corresponding Hylleraas wavefunction. (Right) plot of the TC wavefunction for Li^+^ against the corresponding Hylleraas wavefunction. Both plots show that the 18 parameter TC wavefunction reproduces the Hylleraas wavefunction well.

## TC Energies Using Schmidt–Moskowitz Parameters

Schmidt and Moskowitz have previously found sets of 7, 9, and 17 correlator parameters for first-row atoms via variance minimization.^56^ Following Alavi and co-workers, we shall refer to these sets as SM7, SM9, and SM17, respectively. The correlator has the form in eq 18. For ease of reference, the various terms incorporated in the correlator for SM7, SM9, and SM17 are tabulated in Table 1.

**Table 1:** Summary of the Various Sets of Parameters Used, Where Each Number in the Second Column Has the Form *mno*^a^

set	parameters
SM7	001, 002, 003, 004, 200, 300, 400
SM9	001, 002, 003, 004, 200, 300, 400, 220, 202
SM17	001, 002, 003, 004, 200, 300, 400, 220, 202, 222, 402, 204, 422, 602, 404, 224, 206
SOM8	001, 002, 003, 004, 100, 200, 300, 400
SOM10	001, 002, 003, 004, 100, 200, 300, 400, 220, 202
SOM18	001, 002, 003, 004, 100, 200, 300, 400, 220, 202, 222, 402, 204, 422, 602, 404, 224, 206

a For example, 001 corresponds to the *m* = 0, *n* = 0, and *o* = 1 term, i.e., *r*_*ij*_ term. “SM” refers to Schmidt–Moskowitz parameters while ”SOM” refers to parameters found via SOM minimization.

Using the correlator parameters found by Schmidt and Moskowitz, we solved the TC Hamiltonian for the first-row atoms with a series of correlation-consistent basis sets (cc-pVXZ, X = D, T, Q). This was done by using the corresponding Unrestricted Hartree–Fock orbitals as a starting guess and varying it until self-consistency. The data in Tables 2–4 show that for a fixed set of correlator parameters, the TC total energies of small atoms converge with basis set size. However, the TC energies do not necessarily decrease with an increasing number of parameters used. For example, in atoms from boron through neon, the TC energies increase going from 9 parameters to 17 parameters. This can be understood when we consider the origin of the parameters used. Schmidt and Moskowitz used Slater-type orbitals (STOs) in their optimization studies to obtain the parameters. On the other hand, this work employs Gaussian-type orbitals (GTOs). One major difference between the STOs and GTOs is that the electron-nuclear cusp condition is fulfilled while using STOs but not when using GTOs. Different corrections are therefore required for the Hartree–Fock solutions expressed with different orbital bases, leading to the need for different parameters. Hence, the parameters used in this study may not be optimal.

**Table 2:** Comparison of the TC Total Energies (in Hartrees) Found with the Bi-Variational Approach Using 7 Parameters against Literature and Experimental Values^a^

	cc-pVDZ	cc-pVTZ	cc-pVQZ	SM7^56^	FCIQMC (cc-pVQZ)^35^	experimental^57^
He	–2.8962	–2.9021	–2.9025	–2.8997		–2.9037
Li	–7.4670	–7.4671	–7.4672	–7.4746	–7.4779	–7.4781
Be	–14.6111	–14.6112	–14.6113	–14.6259	–14.6679	–14.6674
B	–24.5740	–24.5756	–24.5764	–24.5946	–24.65417	–24.6539
C	–37.7431	–37.7475	–37.7489	–37.7721	–37.8479	–37.8450
N	–54.4502	–54.4593	–54.4618	–54.5019	–54.5878	–54.5892
O	–74.8659	–74.8849	–74.8659	–74.9469	–75.0630	–75.0673
F	–99.4619	–99.4912	–99.4989	–99.5746	–99.7251	–99.7339
Ne	–128.6119	–128.6528	–128.6640	–128.7689	–128.9297	–128.9376

a The parameters were the same as that used by Schmidt and Moskowitz.^56^ FCIQMC (cc-pVQZ basis) data were found by Alavi and co-workers.^35^ Experimental values were found by Chakravorty and co-workers.^57^

**Table 3:** Comparison of the TC Total Energies (in Hartrees) Found with the Bi-Variational Approach Using 9 Parameters against Literature and Experimental Values^a^

	cc-pVDZ	cc-pVTZ	cc-pVQZ	SM9^56^	FCIQMC^35^	experimental^57^
He	–2.8935	–2.8995	–2.8998	–2.9029		–2.9037
Li	–7.4746	–7.4727	–7.4724	–7.4731		–7.4781
Be	–14.6205	–14.6191	–14.6192	–14.6332		–14.6674
B	–24.6057	–24.6055	–24.6062	–24.6113		–24.6539
C	–37.7592	–37.7632	–37.7644	–37.7956		–37.8450
N	–54.5262	–54.5334	–54.5349	–54.5390		–54.5892
O	–74.9971	–75.0136	–75.0164	–75.0109		–75.0673
F	–99.6589	–99.6873	–99.6920	–99.6685		–99.7339
Ne	–128.8567	–128.8985	–128.9070	–128.8796		–128.9376

a The parameters were the same as those used by Schmidt and Moskowitz.^56^ FCIQMC (cc-pVQZ basis) data have not been previously reported. Experimental values were found by Chakravorty and co-workers.^57^

**Table 4:** Comparison of the TC Total Energies (in Hartrees) Found with the Bi-Variational Approach Using 17 Parameters against Literature and Experimental Values^a^

	cc-pVDZ	cc-pVTZ	cc-pVQZ	SM17^56^	FCIQMC^35^	experimental^57^
He	–2.8959	–2.9020	–2.9023	–2.9036		–2.9037
Li	–7.4770	–7.4766	–7.4765	–7.4768	–7.4785	–7.4781
Be	–14.6304	–14.6300	–14.6283	–14.6370	–14.6675	–14.6674
B	–24.5974	–24.5980	–24.5977	–24.6156	–24.6529	–24.6539
C	–37.7740	–37.7766	–37.7772	–37.8017	–37.8446	–37.8450
N	–54.5022	–54.5099	–54.5116	–54.5456	–54.5884	–54.5892
O	–74.9549	–74.9719	–74.9549	–75.0146	–75.0661	–75.0673
F	–99.5830	–99.6117	–99.6187	–99.6736	–99.7328	–99.7339
Ne	–128.7533	–128.7925	–128.8043	–128.8796	–128.9354	–128.9376

a The parameters were the same as those used by Schmidt and Moskowitz.^56^ FCIQMC (cc-pVQZ basis) data were found by Alavi and co-workers.^35^ Experimental values were found by Chakravorty and co-workers.^57^

Alavi and co-workers have also used correlation-consistent bases in their work on the TC Hamiltonian. However, they are able to find energies in excellent agreement with experimental values (Tables 2–4). We believe that this is due to the effective multi-reference nature of the FCIQMC method such that any errors incurred from using these SM parameters are corrected for by adjusting the weight of each determinant.

## SOM Minimization of Correlator Parameters

### Singlet State Atoms

To improve upon the accuracy of our results, we allowed correlator parameters to vary alongside the orbitals. The correlator parameters were optimized by using SOM minimization. The parameters found from Schmidt and Moskowitz (the set of 7 parameters) were used as a starting guess for the optimization, with an additional *mno* = 001 term to correct for the electron-nuclear cusp conditions (SOM8 in Table 1). The starting guess for the *mno* = 001 term is zero. This set of 8 parameters will be referred to as SOM8. The BFGS algorithm was used for optimization. In practice, we have found it to be useful to optimize the parameters using a two-step SOM minimization procedure where we first keep the orbitals fixed through the optimization process and after the first round of optimization, we perform SOM minimization with orbital relaxation in each iteration of the second optimization cycle. In doing so, we are less likely to get caught in local minima after orbital relaxation. The TC energies found using the optimized parameters are tabulated in Table 5. Closed-shell singlet state atoms were used as examples as it side-steps the possible issue of spin-contamination since we would be able to use a Restricted Hartree–Fock determinant as the reference determinant.

**Table 5:** Comparison of Energies Found after SOM Minimization and the Exact Energies^a^

	HF	SOM8	exact*	difference	correlation energy (%)
^1^S He	–2.8615	–2.8947	–2.9037	0.0090	79
^1^S Be	–14.5730	–14.6663	–14.6674	0.0011	99
^1^S C	–37.6042	–37.7435	–37.7465	0.0030	98
^1^S O	–74.6897	–74.9093	–74.9133	0.0040	98
^1^S Ne	–128.5435	–128.8758	–128.9376	0.0618	84

a The difference (in Hartrees) and the percentage of correlation energy found were similarly reported. *The exact energies of ^1^S C and ^1^S O were deduced from spectroscopic measurements.^58,59^ The exact energies of the other closed shell atoms were taken from experimental values found by found by Chakravorty and co-workers.^57^ The optimized parameters can be found in the Appendix (Table 10). All calculations were run with a cc-pVQZ basis set.

The energies found did not appear to suffer from non-variationality. For the ^1^S states of Be, C, and O, very accurate energies could be found, which recover more than 98% of the correlation energy. While the results for helium and neon were not as encouraging, we note that a highly accurate energy of −2.9037*E*_h_ for helium could be found by using a different starting guess (Table 6). A similarly good result of −2.9033*E*_h_ (see Table 7) can also be found by using the SOM18 set of parameters.

**Table 6:** TC Energies of the Helium Atom in Hartrees for Different Starting Guesses^a^

initial guess	TC energy
*c*_001_ = 0.5, *c*_100_ = +1	–2.8969
*c*_001_ = 0.5, *c*_100_ =–1	–2.8989
*c*_001_ = 0.5, *c*_100_ =–2	–2.9037

a The set of parameters SOM8 was used, with starting guesses of 0 unless otherwise stated in the first column. Starting from SOM8 with *c*_001_ = 0.5, *c*_100_ = −2, and other parameters zero, a highly accurate energy of the helium atom could be found. The optimized parameters can be found in the Appendix (Table 11). All calculations were run with a cc-pVQZ basis set.

**Table 7:** Comparison of Absolute Error in the HF Energy against the Absolute Error in the Energy Found by SOM Minimization^a^

	basis	HF	SOM18	exact	error (HF)	error (SOM)	correlation energy (%)
^1^S H^–^	aug-cc-pVQZ	–0.4878	–0.5231	–0.5278	0.0400	0.0047	88
He	cc-pVQZ	–2.8615	–2.9033	–2.9037	0.0422	0.0004	99
^1^S Li^+^	cc-pCVQZ	–7.2364	–7.2807	–7.2799	0.0435	–0.0008	101
^1^S Be^2+^	cc-pCVQZ	–13.6113	–13.6558	–13.6556	0.0443	–0.0002	100
^1^S B^3+^	cc-pCVQZ	–21.9862	–22.0298	–22.0309	0.0447	0.0011	98
^1^S C^4+^	cc-pCVQZ	–32.3611	–32.4038	–32.4062	0.0451	0.0024	95
^1^S N^5+^	cc-pCVQZ	–44.7360	–44.7779	–44.7814	0.0454	0.0035	92
^1^S O^6+^	cc-pCVQZ	–59.1110	–59.1486	–59.1566	0.0456	0.0080	82
^1^S F^7+^	cc-pCVQZ	–75.4859	–75.5214	–75.5317	0.0458	0.0103	78
^1^S Ne^8+^	cc-pCVQZ	–93.8608	–93.8966	–93.9068	0.0460	0.0102	78

a The absolute error for both HF and SOM minimization methods increases with the magnitude of nuclear charge. The absolute error found from HF is consistently above that of those found from SOM minimization. The optimized correlator parameters found are tabulated in the Appendix (Tables 12 and 13).

In practice, there were a number of local minima found during optimizations depending on the starting guess and hence a number of possible energies can in principle be found. A sample of possible solutions for helium are tabulated in Table 6. This suggests that the starting guess is very important to obtain the right correlator parameters and one should be cautious about the parameters found from such an optimization.

In the case of neon, the use of a larger set of parameters (SOM18) gave a non-variational energy of −129.0019*E*_h_. This demonstrates the possibility of obtaining non-variational energies and highlights the potential pitfalls of using SOM minimization to obtain correlator parameters.

### Helium-like Systems

Encouraged by the possibility of highly accurate energies using SOM minimization, we examined the approach on a series of helium-like systems (Table 7). To find the correlator parameters for this series, we used the set of 17 parameters from Schmidt and Moskowitz and with an additional *mno* = 001 term as a starting guess for the helium atom and performed SOM minimization to obtain the optimized parameters (SOM18). These optimized parameters were then used as starting guesses for each of these ions.

We found that it was important to use an augmented basis set (aug-cc-pVQZ) for the negatively charged hydride anion as more diffuse functions are required to describe the expanded orbitals. In contrast, a basis optimized for describing core–core correlations (cc-pCVQZ) was found to be useful to describe the contracted orbitals in cations.

For comparison, the same calculations were performed with a cc-pVQZ basis set and the results are tabulated in Table 8. The use of the cc-pVQZ basis increased the absolute error from SOM minimization and the TC energies found were mostly non-variational. This shows that the choice of basis set is imperative to the accuracy of the TC method.

**Table 8:** Comparison of Absolute Error in the HF Energy against Those Found by SOM Minimization in the Cc-pVQZ Basis^a^

	basis	HF	SOM18	error (HF)	error (SOM)
^1^S H^–^	cc-pVQZ	–0.4735	–0.5078	0.0543	0.0200
^1^S Li^+^	cc-pVQZ	–7.2364	–7.2872	0.0435	–0.0073
^1^S Be^2+^	cc-pVQZ	–13.6113	–13.6652	0.0443	–0.0096
^1^S B^3+^	cc-pVQZ	–21.9862	–22.0455	0.0447	–0.0146
^1^S C^4+^	cc-pVQZ	–32.3611	–32.4231	0.0451	–0.0169
^1^S N^5+^	cc-pVQZ	–44.7360	–44.8001	0.0454	–0.0187
^1^S O^6+^	cc-pVQZ	–59.1108	–59.1780	0.0458	–0.0214
^1^S F^7+^	cc-pVQZ	–75.4857	–75.5548	0.0460	–0.0231
^1^S Ne^8+^	cc-pVQZ	–93.8605	–93.9309	0.0463	–0.0241

a The absolute error from SOM minimization is significantly higher than those found in Table 7. Most of the TC energies found are also non-variational.

Using the appropriate basis sets for cations and anions, we found that the absolute error from SOM minimization is lower than that for HF for each ion (Table 7). From H^–^ through N^5+^, SOM minimization recovers a large proportion of the correlation energy. From O^6+^ through Ne^8+^, the percentage of correlation energy recovered drops considerably. This is likely due to the highly contracted nature of the 1s orbitals in these highly charged cations and a bigger basis with more contracted basis functions would be required to more accurately describe the electron correlation. However, it is possible that a better starting guess could similarly improve the correlation energy recovered.

It is also gratifying to note that most of the energies found from SOM minimization using appropriate basis sets do not exhibit non-variationality. Li^+^ and Be^2+^ were found to have non-variational energies . However, the error is small and within chemical accuracy (within ∼0.0016*E*_h_).

### Ionization Energies

A further investigation of the TC method could be made by determining the ionization energies within the SOM minimization framework. For consistency, we shall use the cc-pCVQZ basis set for the following ionization processes (Table 9): Be → Be^2+^, C → C^4+^, and O → O^6+^.

**Table 9:** Comparison of Ionization Energies Found in HF against Those Found by SOM Minimization in the Cc-pCVQZ Basis^a^

	HF (atom)	HF (ion)	SOM18 (atom)	SOM18 (ion)	HF I.E.	SOM18 I.E.	Exact I.E.
Be	–14.5730	–13.6113	–14.6355	–13.6556	0.9617	0.9799	1.0118
C	–37.6043	–32.3611	–37.7148	–32.4062	5.2432	5.3086	5.3403
O	–74.6897	–59.1110	–74.8588	–59.1566	15.5787	15.7022	15.7576

a The error from SOM minimization is smaller than that found by HF. The ionization energies are found by taking the energy difference between the closed shell singlet atoms and that of the two-electron ion. The optimized correlator parameters found are tabulated in the Appendix (Table 14).

It is observed that the SOM18 energies for the atoms are higher than that found in Table 5. This is an indication that the near-exact energies found with 8 parameters previously may have been fortuitous. The comparatively poorer description of the atoms as compared to the ions consequently leads to poorer ionization energies. While the predicted ionization energies are an improvement from Hartree–Fock levels of theory, they exhibit appreciable (∼30 mE_h_) errors when compared to the exact ionization energies. This shows that while SOM minimization could in principle provide near-exact energies (both absolute and relative), the optimization procedure may be difficult in practice.

## Graphical Analysis of Correlation

### Electron–Electron Cusp

To better understand the effects of the electron–electron and electron–nucleus terms in the correlator, we studied the effects of various Jastrow factors e^τ^ on a Hartree–Fock solution Φ_HF_ of a helium atom. We first attempted to study the effects of varying the angle θ between two electrons confined to the same electron–nucleus distance (Figure 1, left). Using the correlator  and the Slater determinant found with a Hartree–Fock calculation with a cc-pVQZ basis, the TC wavefunction e^τ^Φ_HF_ was plotted as a function of θ (Figure 2) for varying values of the parameter *c*. The 6-term Hylleraas wavefunction,^60^ which represents a good approximation to the exact wavefunction of helium, is also plotted for comparison.

For ease of reference, the Hylleraas wavefunction for He is given by^60^
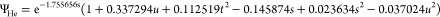
39

where *s* = |***r***_1_| + |***r***_2_|, *t* = |***r***_1_| – |***r***_2_|, and *u* = |***r***_1_ – ***r***_2_|.

The Jastrow factor’s introduction of electron–electron cusps to the Hartree–Fock solution can be seen from Figure 2. The shape of the electron–electron cusp gets increasingly similar to that of the Hylleraas wavefunction as the coefficient increases from 0.1 to 0.5. This supports the use of  to correct for the electron–electron cusps. The coefficient  is fixed in the TC calculations, which therefore necessitates the need for higher order terms in *r*_*ij*_ to correct for the depth of the cusp.

### Electron–Nuclear Cusp

To examine the effect of the electron–nuclear cusp, we use the special case whereby the nucleus and an electron are constrained to be 1.26 Bohr away and the other electron is free to move along the line defined by them (Figure 1, right). We use a different correlator  where *r* is the variable electron–nuclear distance and *r*_12_ = *r* – *R* is the electron–electron distance and vary the value of parameter *c*. The Hartree–Fock wavefunction most closely matches that of the Hylleraas wavefunction at the nucleus while the function with *c* = −1 is more similar further away from it (Figure 3).

For comparison, the function with *c* = −2 was plotted as *c* = −2 and is what would be expected from a simple application of Kato’s cusp conditions. This illustrates that the coefficient *c* need not be equal to the negative of the nuclear charge, −*Z*, as the electron–nuclear interaction term in the Jastrow factor affects the overall wavefunction and not only at the cusp. Performing SOM minimization with this correlator, we found a value of *c* = −1.12, and the TC wavefunction with *c* = −1.12 is plotted. The plot shows good agreement with the Hylleraas wavefunction (Figure 4).

A similar series of calculations were attempted for Li^+^. The Hylleraas wavefunction for Li^+^ is given by
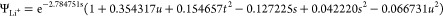
40

From Figure 5, it can be seen that the Hartree–Fock wavefunction is very similar to that of the Hylleraas wavefunction near the nucleus. At regions further from the nucleus, the *c* = −1 wavefunction most closely resembles the Hylleraas wavefunction. SOM minimization gave *c* = −3.79, but in this case, we were unable to reproduce the Hylleraas wavefunction (Figure 6). There are several reasons why such a discrepancy can exist.

First, resolution of identity is an approximation, which may not be valid depending on the size of the basis set used.

Second, the Hartree–Fock wavefunction resembles the Hylleraas wavefunction well. While the addition of a Jastrow factor to it can provide a better description of the electron–nuclear cusp, it comes at the cost of affecting other parts of the wavefunction. More terms in the correlator may need to be added to more accurately describe the TC wavefunction at different points in space.

To address the latter point, the TC wavefunctions for He and Li^+^ found using the SOM18 set of parameters (Table 7) were plotted against the respective Hylleraas wavefunctions (Figures 7 and 8). From Table 7, it can be observed that the TC energies are very close to the exact energies, and this suggests that the TC wavefunctions should look similar to that of the Hylleraas wavefunction. This is reflected graphically in the depiction of the electron–electron cusp (Figure 7) and electron-nuclear cusp (Figure 8), where each of the TC wavefunctions agree well with the corresponding Hylleraas wavefunction. The main difference between the TC wavefunction and Hylleraas wavefunctions appears to be the description of electron–electron interactions at larger electron–electron distances, which may hint at the use of a differently scaled form of the correlator to account for longer range effects. Overall, the plots show that SOM minimization can get highly accurate wavefunctions given sufficiently many correlator parameters, supporting the utility of SOM minimization when appropriate starting guesses are used.

## Conclusions

A self-consistent method for solving the non-self-adjoint TC Hamiltonian has been implemented successfully. We have found that it is possible to obtain highly accurate energies of some first row atoms from this approach. The correlator parameters found in the literature are not optimized for the Gaussian orbital basis used in this current study and had to be re-optimized through a method we refer to as SOM minimization. This allowed us to find optimized parameters for any system, in principle. However, the optimization of multiple parameters is challenging and in practice, we have found it to be useful to optimize the parameters using a two-step SOM minimization procedure. SOM minimization has been found to give good energies for the first row atoms. However, the percentage of correlation energy recovered has been found to decrease with increased nuclear charge across a series of helium-like ions. We believe that this is due to the inability of the basis set to accurately describe highly charged cations, and a custom basis with more contracted basis functions would be helpful to describe the correlation in these systems. The analysis on ionization energies of select atomic systems suggests that there is still room to improve upon the SOM minimization method. We leave this for future work. A graphical analysis has also been done to illustrate the effects of some correlator terms on the overall wavefunction and demonstrated the importance of including higher-order correlator terms in the Jastrow factor to give a more accurate wavefunction.

Thus far, SOM minimization has been attempted for closed-shell systems. Further work has to be done on open-shell systems where there is a possibility of spin-symmetry breaking, leading to an unphysical wavefunction. This could in principle be tackled with NOCI,^62−64^ which forms the basis for future work. SOM minimization should also be attempted on larger systems to test if the method works more generally. However, as pointed out by Alavi and co-workers,^35^ memory use is a bottleneck in TC calculations. This can be challenging especially with the need to use large basis sets for SOM minimization as it relies on the resolution-of-the-identity approximation. A possible solution would be to use an auxiliary basis set instead, which would allow us to use a smaller basis set to represent the Slater determinant but a large auxiliary basis to satisfy the resolution-of-the-identity approximation. Developing methods to make SOM minimization more robust for calculating near-exact energies for a range of systems would also be desirable, and we hope that this work will inspire further efforts in this area.

## Appendix A

### TC Hamiltonian

#### Expansion of Commutator Terms

We first separate the many-electron electronic Hamiltonian into a kinetic energy  and potential energy  term

41

 is multiplicative and hence commutes with τ in the commutator , that is

42

The commutator terms in eq 5 will be evaluated term by term as follows

43

We can now make the identification

44

45

Therefore

46

Since  is a multiplicative term, higher-order commutators of the form  vanish.

Given that τ = ∑_*i*<*j*_*u*(***r***_*i*_, ***r***_*j*_), we can further expand eq 6.

47

where we used the symmetry *u*(***r***_*i*_, ***r***_*j*_) = *u*(***r***_*j*_, ***r***_*i*_). We therefore find

48

where we relabeled *a* by *j* and used the symmetry of *u* in the last line.

We similarly find

49

Finally

50

Substituting these commutator terms back into eq 5 recovers eq 6.

#### Lagrangian Approach to the TC Hamiltonian

We show here that the method of Lagrange multipliers can be used to derive the effective TC Hamiltonian.

51

In the last line, we have renamed the *n*-electron operators by the . This is for brevity of notation and the understanding that the mathematics after is concerned only with the number of electrons the operators act upon. Taking an infinitesimal change of the Lagrangian with respect to variation of the spin-orbitals

52

We shall analyze each term of the above expression in turn. We will adopt the shorthand

We assume that the bra always contains molecular orbitals from the set {ψ_*i*_} and that the ket always contains molecular orbitals from the set {ϕ_*i*_}.

##### One-Electron Term

53

.

##### Two-Electron Term

54

where we have used the permutation symmetry of the integrals, e.g., .

##### Three-Electron Term

55

.

##### Lagrangian Differential

56

where we have defined the following

57

We use the shorthand such that

*S*_*n*_ being the permutation group of *n* elements (eq 17). Similarly

58

We seek  for any arbitrary changes of ψ and ϕ independently. Hence,  and  independently.

##### Effective TC Hamiltonian

Consider the condition .

59

Rewriting the expression more explicitly in integral form

60

61

Since the expression holds for any δψ_*i*_^*^, the terms in the square bracket must be zero. Hence

62

for all *i*. Rearrangement of the equation recovers the equation given in eq 14.

## Appendix B

### Correlator Parameters

#### Correlator Parameters for Closed-Shell Atoms (SOM8)

The correlator parameters for closed-shell ions (Table 5) found from using SOM minimization on a set of 8 parameters are tabulated in Table 10.

**Table 10:** Correlator Parameters for Closed-Shell Atoms Found from Using SOM Minimization^a^

*m*	*n*	*o*	He	Be	C	O	Ne
0	0	1	0.50000	0.50000	0.50000	0.50000	0.50000
0	0	2	0.42476	0.21162	0.03755	–0.45608	–0.75272
0	0	3	–0.23302	0.30937	–0.18384	0.67271	1.31436
0	0	4	0.28445	–0.21062	0.42119	0.04159	–0.36159
1	0	0	0.00151	0.01082	–0.00952	–0.02441	–0.00979
2	0	0	–0.16865	–0.11872	–0.12690	–0.12667	–0.14499
3	0	0	–0.34421	–0.17257	–0.05827	–0.01992	–0.00973
4	0	0	–0.54727	0.16579	0.08346	0.02964	0.08552

a These parameters are used to obtain the TC energies in Table 5.

#### Correlator Parameters for Helium from Different Initial Guesses (SOM8)

The correlator parameters for helium (Table 6) found from using different initial guesses are tabulated in Table 11.

**Table 11:** Correlator Parameters for Helium Found from Using SOM Minimization with Different Initial Guesses^a^

*m*	*n*	*o*	*c*_100_ = +1	*c*_100_ =–1	*c*_100_ =–2
0	0	1	0.50000	0.50000	0.50000
0	0	2	0.29627	0.29152	0.29071
0	0	3	0.12627	0.14407	0.15048
0	0	4	0.04485	0.06602	0.07330
1	0	0	0.99565	–1.00073	–1.99929
2	0	0	–0.00338	–0.00177	–0.00116
3	0	0	–0.00200	–0.00159	–0.00134
4	0	0	–0.00110	–0.00101	–0.00100

a These parameters are used to obtain the TC energies in Table 6.

#### Correlator Parameters for Helium-like Ions (SOM18)

The correlator parameters for helium-like ions found from using SOM minimization on a set of 18 parameters are tabulated in Tables 12 and 13. The parameters for helium were used as a starting guess for the helium-like systems in Table 7.

**Table 12:** Correlator Parameters for Helium-like Ions (H^–^ to B^3+^) Found from Using SOM Minimization

*m*	*n*	*o*	H^–^	He	Li^+^	Be^2+^	B^3+^
0	0	1	0.50000	0.50000	0.50000	0.50000	0.50000
0	0	2	0.17646	0.10188	0.04101	–0.02237	–0.09254
0	0	3	–0.33069	–0.38197	–0.40395	–0.42278	–0.44691
0	0	4	0.99262	0.95942	0.95251	0.94670	0.93836
1	0	0	0.00594	–0.00015	0.00225	0.00645	0.01269
2	0	0	0.23633	0.23188	0.23339	0.23499	0.23692
3	0	0	–0.44818	–0.45048	–0.44978	–0.44932	–0.44888
4	0	0	0.82844	0.82766	0.82795	0.82804	0.82811
2	2	0	–4.16199	–4.15465	–4.15276	–4.15252	–4.15303
2	0	2	0.84241	0.80798	0.80157	0.79821	0.79362
2	2	2	10.19802	10.19694	10.19694	10.19683	10.19663
4	0	2	–4.95213	–4.96235	–4.96281	–4.96305	–4.96336
2	0	4	–1.34054	–1.35616	–1.35651	–1.35712	–1.35804
4	2	2	–5.91087	–5.90919	–5.90919	–5.90931	–5.90940
6	0	2	0.90672	0.90347	0.90345	0.90341	0.90338
4	0	4	5.51230	5.50742	5.50747	5.50736	5.50726
2	2	4	–0.03148	–0.03160	–0.03156	–0.03163	–0.03170
2	0	6	–1.04601	–1.05188	–1.05165	–1.05186	–1.05208

**Table 13:** Correlator Parameters for Helium-like Ions (C^4+^ to Ne^8+^) Found from Using SOM Minimization

*m*	*n*	*o*	C^4+^	N^5+^	O^6+^	F^7+^	Ne^8+^
0	0	1	0.50000	0.50000	0.50000	0.50000	0.50000
0	0	2	–0.16520	–0.24505	–0.21726	–0.28297	–0.40323
0	0	3	–0.47290	0.92623	–2.85437	–2.72267	–2.81519
0	0	4	0.92969	0.02574	–0.22549	–0.03555	–0.07534
1	0	0	0.02024	0.23972	0.73292	0.78108	0.63683
2	0	0	0.23880	–0.44851	0.42552	0.41999	0.37976
3	0	0	–0.44855	0.82809	–0.40369	–0.40880	–0.41889
4	0	0	0.82814	–4.15395	0.83915	0.83706	0.83451
2	2	0	–4.15379	–4.15465	–4.27033	–4.24468	–4.24557
2	0	2	0.78910	0.78741	0.17596	0.29033	0.25548
2	2	2	10.19648	10.19646	10.18118	10.18753	10.18630
4	0	2	–4.96357	–4.96347	–5.00535	–4.99058	–4.99174
2	0	4	–1.35874	–1.35859	–1.47410	–1.43261	–1.43795
4	2	2	–5.90944	–5.90945	–5.91112	–5.91019	–5.91031
6	0	2	0.90337	0.90340	0.89998	0.90163	0.90157
4	0	4	5.50723	5.50727	5.49931	5.50322	5.50289
2	2	4	–0.03174	–0.03174	–0.03402	–0.03279	–0.03297
2	0	6	–1.05219	–1.05213	–1.06807	–1.06012	–1.06121

**Table 14:** Correlator Parameters for Closed-Shell Singlet States for Be, C, and O Found from Using SOM Minimization

*m*	*n*	*o*	Be	C	O
0	0	1	0.50000	0.50000	0.50000
0	0	2	–0.01703	–0.15642	–0.31187
0	0	3	–0.62360	–0.50658	–0.83160
0	0	4	1.38434	1.01781	1.16077
1	0	0	–0.00141	–0.00107	0.00039
2	0	0	0.38138	0.21553	0.04824
3	0	0	–0.40286	–0.02800	0.09102
4	0	0	1.38158	0.28159	0.11887
2	2	0	–5.23645	–3.72201	–3.34370
2	0	2	0.66005	–0.27993	1.01888
2	2	2	13.83385	13.13639	10.00136
4	0	2	–5.39819	–1.02462	–2.20107
2	0	4	–2.65847	–1.25441	–2.46406
4	2	2	–2.13248	–4.62164	–5.46127
6	0	2	–0.80519	–1.32252	–0.08974
4	0	4	8.42138	5.22751	5.47474
2	2	4	–4.73743	–4.17387	–0.24798
2	0	6	–1.49405	–1.65145	–1.70128
